# A novel adiposity index as an integrated predictor of cardiometabolic disease morbidity and mortality

**DOI:** 10.1038/s41598-018-35073-4

**Published:** 2018-11-13

**Authors:** Yousung Park, Nam Hoon Kim, Tae Yeon Kwon, Sin Gon Kim

**Affiliations:** 1 Department of Statistics, Korea University, Seoul, South Korea; 2 Division of Endocrinology and Metabolism, Department of Internal Medicine, Korea University College of Medicine, Seoul, South Korea; 3 Department of International Finance, Hankuk University of Foreign Studies, Yongin-si, Gyeonggi-do South Korea

## Abstract

We propose a new anthropometric index, weight-adjusted-waist index (WWI), to assess adiposity by standardizing waist circumference (WC) for weight. WWI, calculated as WC (cm) divided by the square root of weight (kg) (cm/√kg), was measured from 465,629 subjects in the Korean nationwide cohort (2008–2013). Cox regression analysis was used to compare WWI with BMI, WC, waist-to-height ratio (WHtR), and a body shape index (ABSI) for cardiometabolic morbidity and mortality risk in diagnostic and prognostic prediction models. For incident hypertension, type 2 diabetes and cardiovascular disease (CVD), BMI had the strongest predictive power, followed by WWI and WC. However, WWI showed the best predictive performance for CVD mortality. Also, a linear positive association between adiposity indices and cardiovascular and all-cause mortality was only shown in WWI and ABSI, not BMI, WC and WHtR which showed inverse J-shaped patterns. In the test of joint effects of each index, WWI combined with BMI was the strongest in both diagnostic and prognostic models. WWI is a unique adiposity index that shows linear positive association with both cardiometabolic morbidity and mortality. It also predicts incident cardiometabolic disease, cardiovascular and all-cause mortality risk with excellence in predictive power, especially when combined with BMI.

## Introduction

Obesity, defined as excess body fat, is a well-known risk factor for various chronic diseases including hypertension, type 2 diabetes, dyslipidemia, non-alcoholic fatty liver disease, and cardiovascular disease (CVD)^1^. Body mass index (BMI) is the most widely used measure of obesity. The prevalence of obesity, defined as a high BMI, has been increasing worldwide along with obesity-related disorders^2,3^. Much evidence indicated a linear association between BMI and risk of hypertension, type 2 diabetes, and CVD^4,5^. However, inconsistent or inverse associations between BMI and mortality in various population have resulted in the “obesity paradox”^6–8^. Several explanations for this paradox include survival or selection bias, treatment bias, or confounding effects of major risk factors^9,10^. On the other hand, some investigators have indicated the limitation of BMI as a true measure of obesity, as it does not differentiate between lean mass and fat mass and is thus limited by differences of body adiposity for a given BMI level across age, gender, and race^11,12^. In a meta-analysis that pooled 32 studies, BMI identified half of 32,000 individuals with excess body fat as normal^13^.

Waist circumference (WC) has been proposed as a more accurate body adiposity index for the prediction of obesity-related disorders than BMI, because it has excellent correlations with abdominal fat imaging and high association with CVD risk factors and mortality^14–17^. However, WC is highly correlated with BMI, and is thus limited as an independent measure of BMI. Moreover, the phenomenon of obesity paradox has also been identified when obesity was measured by WC^18^. Another study has shown that high WC predicted increased mortality among patients with acute myocardial infarction only in consideration with BMI^19^. Other indices such as waist-to-height ratio (WHtR) and a body shape index (ABSI) defined by WC/(*BMI*^2/3^*Height*^1/2^) have been suggested as alternative indicators of obesity with some advantages than traditional indices. WHtR was better than BMI in association with diabetes and CVD risks^20,21^. ABSI is a new body shape index which has been developed based on WC divided by its regression fit on weight and height so that ABSI is minimally correlated with weight, height, and BMI. ABSI was found to be more closely associated with mortality risk than BMI and WC^22^. However, WHtR also had a strong correlation with BMI, thus, it was not free from the influence of BMI, and had a weaker association with mortality than BMI. In contrast, ABSI was uncorrelated with BMI, but several studies indicated that it is a less reliable indicator of cardiometabolic risk factors than BMI or WC^23,24^.

Therefore, the limitations of traditional and newer anthropometric indicators suggest the need for an integrated adiposity index to have an ability to predict both cardiometabolic disease morbidity and mortality. We herein propose a new adiposity index termed the “weight-adjusted-waist index” (WWI) that is a standardized WC for weight, and aimed to validate the new index for the association with obesity-related disorders, cardiovascular mortality and all-cause mortality.

## Methods

### Derivation of WWI

We wanted an adiposity index representing waist circumference, having weak correlation with BMI to alleviate the obesity paradox of BMI for death, and having a negative correlation with height to differentiate the effect of height on the same waist. This could be accomplished by adjusting waist only for weight. When the BMI was first proposed, it was calculated by standardizing weight for height. Namely, the BMI was obtained by regressing the logarithm of weight on the logarithm of height to remove the effect of height on weight^25^ (the correlation between weight and height was 0.666, but that between BMI and height was as low as 0.072 in our data set). With the same concept, we proposed an index which standardizes waist circumference for body weight by the least squared regression of the logarithm-transformed WC on the logarithm-transformed weight as given by

$$\mathrm{ln}({\rm{WC}})={\beta }_{0}+{\beta }_{1}\,\mathrm{ln}({weight})+{\rm{\varepsilon }}{\rm{.}}$$

The estimated ${\beta }_{1}$ was 0.494 (p-value < 0.0001) that is close to 0.5. Thus, ${\rm{In}}({\rm{WC}})-0.5{\rm{In}}({\rm{weight}})=1{\rm{n}}\frac{{wc}}{\sqrt{weight}}$ $\mathrm{ln}\,\frac{wc}{\sqrt{weight}}$ became an estimate of ${\beta }_{0}+{\rm{\varepsilon }}$ so that $\frac{wc}{\sqrt{weight}}$ is almost uncorrelated with *weight* (the correlation was −0.025), by the characteristics of the least squared regression model^24^. We define the weight-adjusted-waist index (WWI) as $\frac{wc}{\sqrt{weight}}$ whose mean values were 10.0 (±0.63) for males and 10.1 (±0.86) for females.

### Study population

The Korean National Health Insurance Cohort (NHIS) study (2002–2013) is a population-based longitudinal study consisting of about one million Koreans, a representative 2.2% sample of the national population data. The NHIS data are composed of demographic information, anthropometric measures including body weight (kg) and height (m), medical and pharmacy records, health examination data, and death records. Annually, 10∼15% of the cohort population received health examinations. Since WC (cm) had been measured from 2008, our data-set included only the subjects who had ever undergone health examinations from 2008 to 2013. More detailed information has been given in previous publications^6,26^.

The comparison of a new adiposity index with existing indices was done by model fit and predictive accuracy of incident cardiometabolic diseases including hypertension and type 2 diabetes and of cardiovascular and all-cause mortality. For the mortality prediction, we excluded subjects with pre-existing cancer or CVD before 2008 to avoid a possible confounding effect of those conditions on mortality. For each disease prediction, we also excluded those with corresponding preexisting diseases including hypertension, type 2 diabetes and CVD before 2008 when the specific disease was the risk of interest. For diagnostic data setting, among total 468,981 subjects, 5,105 subjects were additionally deleted for all-cause death and 354 subjects for CVD death due to missing information of explanatory variables. Accordingly, the total number of participants ranged from 425,917 to 465,627 depending on the study risks: 460,876 subjects with 5,469 deaths for all-cause death, 465,627 with 718 deaths for CVD death, 425,917 with 40,334 incidents in 2008 for hypertension, 442,532 with 23,808 incidents in 2008 for type 2 diabetes, 463,797 with 21,984 incidents in 2008 for CVD. The mean follow-up duration for all-cause death was 5.61 (±0.45) years. For prognostic data setting, there were 167,203 subjects who had physical examinations at year 2008. Among them, 2,954 all-cause deaths and 388 CVD deaths occurred until 2013. Prognostic prediction models were applied to this data set for evaluating the effect of adiposity indices on death. The mean follow-up for the prognostic model was 5.96 (0.383) years. All data from the NHIS cohort do not involve any personally identifiable data such as name and personal ID. Thus, NHIS approved the cohort study without informed consent from each person. This study was approved by the institutional review board of Korea University Anam Hospital (IRB number: ED14188).

### Identification of cause of death, disease status, and confounding variables

Causes of death were classified by the International Classification of Disease, Tenth Revision (ICD-10). The data and causes of death of each individual were recorded in their medical records by physicians, and all death records were included in the NHIS data. CVD was identified by the disease codes (I20~I25 and I60-I69). Type 2 diabetes and hypertension were identified by the disease codes (E11~E14, N083, I792, G590, G632, G990, H360, and M142 for type 2 diabetes and I10~I13 and I15 for hypertension) or laboratory data from health examination (fasting plasma glucose level (≥126 mg/dl) for type 2 diabetes, systolic blood pressure (≥140 mmHg) or diastolic blood pressure (≥90 mmHg) for hypertension). Other laboratory variables included in the Cox regression models were hemoglobin, alanine aminotransferase, and gamma-glutamyltransferase. History of smoking (current, former, or never), alcohol consumption (≥3 times/week,≤2 times/week, or never), physical activity (≥3 times/week,≤2 times/week, or never), and socioeconomic status (SES, high 30%, middle 40%, or low 30%) and individual age and sex were also included in the analysis.

### Categorization of each adiposity index

The primary objective of this study was to compare the association between adiposity indices and obesity-related diseases or mortality risk, as well we wanted to show the association patterns or shapes. The established 4 or 5 categories of BMI is useful for clinical application, however, those categorization was limited to display the association patterns. Thus, we used more detailed categorization of BMI and corresponding categorization of each indices. BMI was categorized into 10 groups: <18.5, 18.5–20, 20–21.5, 21.5–23, 23–25, 25–26.5, 26.5–28, 28–30, 30–32.5, ≥32.5 kg/m^2^, where the fifth group (23–25) was the reference group. The 10 groups consisted of 3.99%, 8.21%, 13.50%, 17.71%, 24.53%, 13.87%, 8.65%, 5.81%, 2.67%, and 1.06%, respectively. WC, WHR, ABSI, and WWI were also categorized into 10 groups so that the distribution of the 10 groups was approximately the same as that of BMI for fair comparisons of prediction ability (Table 1).

**Table 1:** Ten groups of adiposity indices with the 5^th^ group as the reference.

Group	1	2	3	4	5	6	7	8	9	10
BMI $(\frac{{\rm{kg}}}{{{\rm{m}}}^{2}})$	<18.5	18.5~20	20~21.5	21.5~23	23.5~25	25~26.5	26.5~28	28~30	30~32.5	≥32.5
WC(cm)	<64	64~68.8	68.8~73.9	73.9~78.6	78.6~84.8	84.8~88.8	88.8~92.2	92.2~97.4	97.4~103	≥103
WHtR $(\frac{{\rm{cm}}}{{\rm{m}}})$	<0.4	0.4~0.43	0.43~0.45	0.45~0.48	0.48~0.51	0.51~0.54	0.54~0.56	0.56~0.60	0.60~0.63	≥0.63
ABSI $(\frac{cm\,\ast \,k{g}^{5/6}}{{m}^{2/3}})$	<6.95	6.95~7.23	7.23~7.49	7.49~7.72	7.72~8.04	8.04~8.26	8.26~9.46	8.46~8.77	8.77~9.13	≥9.13
WWI $(\frac{{\rm{c}}{\rm{m}}}{\surd kg})$	<8.83	8.83~9.22	9.22~9.56	9.56~9.89	9.89~10.34	10.34~10.72	10.72~11.05	11.05~11.51	11.51~12.11	≥12.11

### Statistical Analysis

Pearson’s partial correlation analysis was carried out among the anthropometric indicators adjusting for age and sex. Each subject had multiple health examination records as he/she could have undergone several health examinations during the study period of 2008–2013. To take into account the time-dependent nature of health records and such multiple records of each subject in the analysis, we used the counting process formulations in the Cox regression model. The counting process formulation is a data rearranging method based on the time interval of health examination so that multiple health records of an individual can be represented by multiple observations allocated to non-overlapping time intervals for different health examinations. The average number of health records per participant was 2.14 (1.45) times. The Cox regression applied to these multiple health records of an individual belongs to a diagnostic prediction model. We were also interested in how adiposity indices measured at year 2008 as the base line contribute to the prediction ability of death for certain future time periods. To do this, we applied prognostic prediction models to these data consisted of single heath record per individual at 2008.

In the diagnostic prediction model, we computed likelihood ratio (LR) test statistics to measure partial contribution of adiposity indices to model fit, and C statistics to examine overall measures of predictive accuracy from the models with different adiposity indices as predictors associated with the risks of death and diseases. On the other hand, in the prognostic prediction model, we computed LR, AUC (area under ROC) at three different future time points denoted by year 1, year 3, and year 5 for the predictive accuracy at 1, 3, and 5 years later after 2008, and IAUC (integrated AUC) and C statistics for overall predictive accuracy of prognostic prediction models. The larger the LR, the more contribution the adiposity index for model fit, and the closer the AUC, IAUC, or C is to 1, the better predictive accuracy the model used a specific adiposity index. All methods were performed in accordance with the ethical standards of the institutional and national research committee and with the 1964 Helsinki declaration. All statistical analyses were performed using SAS version 9.4.

## Results

Table 2 displays the mean of each adiposity index for the living and dead persons during 2008–2013. The total number of cohort participants was 460,878 for the all-cause mortality analysis (227,598 men with 3,397 deaths and 233,278 women with 2,072 deaths) and 465,627 in CVD mortality analysis (230,585 men with 410 CVD deaths and 235,042 women with 308 deaths).

**Table 2:** Means values of adiposity indices for all-cause and cardiovascular mortality (values in parentheses are standard deviations).

	Sex		BMI	WC	WHtR	ABSI	WWI	Age
All-cause mortality	Male (n = 227,598)	survivors	24.3 (3.08)	83.9 (7.88)	0.49 (0.05)	10.0 (0.62)	7.91 (0.44)	46.3 (13.2)
		decedents	22.6 (3.29)	82.8 (8.47)	0.50 (0.05)	10.6 (0.76)	8.34 (0.59)	66.5 (12.8)
	Female (n = 233,278)	survivors	23.1 (3.38)	76.0 (8.99)	0.49 (0.06)	10.1 (0.86)	7.71 (0.55)	48.2 (14.5)
		decedents	22.9 (3.89)	79.4 (10.1)	0.53 (0.07)	11.1 (1.11)	8.33 (0.77)	71.3 (13.7)
CVD mortality	Male (n = 230,585)	survivors	24.3 (3.08)	83.9 (7.88)	0.49 (0.05)	10.0 (0.63)	7.91 (0.45)	46.4 (13.3)
		decedents	23.0 (3.36)	84.0 (8.83)	0.51 (0.05)	10.7 (0.80)	8.37 (0.62)	68.2 (12.0)
	Female (n = 235,042)	survivors	23.1 (3.39)	76.0 (8.99)	0.49 (0.06)	10.1 (0.86)	7.71 (0.56)	48.3 (14.5)
		decedents	22.9 (3.85)	79.8 (10.2)	0.54 (0.07)	11.3 (1.09)	8.42 (0.77)	75.2 (10.2)

**Figure 1:**
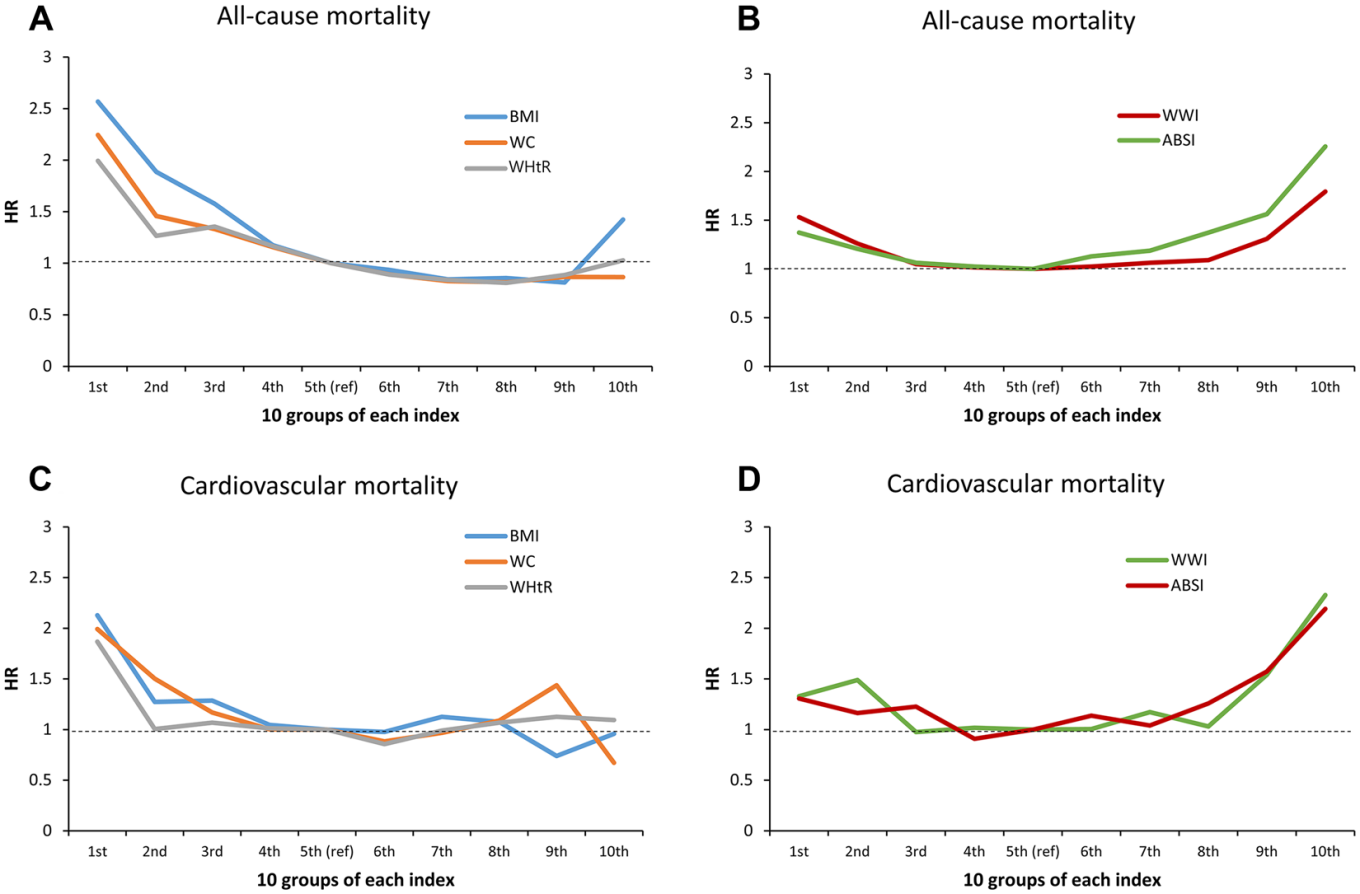
Hazard ratio of 10 groups of each adiposity index on all-cause (**A**,**B**) and cardiovascular mortality (**C**,**D**) (marginal effect).

Mean BMI was unexpectedly higher in survivors than decedents regardless of sex in both analyses. This partly explains the obesity paradox of BMI as illustrated in Fig. 1. On the other hand, the mean values of WHtR, ABSI, and WWI were lower in survivors than decedents. We noted that mean BMI, WC and ABSI were higher in males than in females, whereas mean WHtR and WWI were slightly lower in males than in females (Table 2).

Pearson’s partial correlation analysis was carried out for the each adiposity index (Table 3). BMI was strongly correlated with WC and WHtR, weakly correlated with WWI, and negatively correlated with ABSI. WWI was strongly correlated with ABSI (r = 0.898) and had stronger correlations with WC and WHtR than ABSI, indicating that WWI is more representative for waist-related-indicators than ABSI. Height is not correlated with BMI and ABSI, positively correlated with WC, and negatively correlated with WWI more than WHtR, meaning that WWI differentiated the effect of height on the same WC.

**Table 3:** Pearson correlations among adiposity indices adjusted for age and sex.

	BMI	WC	WHtR	ABSI	WWI	Weight	Height
BMI	1	0.808	0.81	−0.102	0.249	0.888	−0.015
WC		1	0.942	0.472	0.674	0.802	0.171
WHtR			1	0.471	0.776	0.651	−0.163
ABSI				1	0.898	−0.08	0.022
WWI					1	0.11	−0.254

### Adiposity index and mortality risks

#### Diagnostic prediction model

Table 4 provides likelihood ratio test statistics between diagnostic prediction models with and without specific adiposity indices as predictors and C statistics for the diagnostic prediction models with different adiposity indices for all-cause and CVD mortality. The regression models include all confounding variables other than the adiposity index: age, sex, systolic blood pressure, fasting glucose, hemoglobin, alanine aminotransferase, gamma-glutamyltransferase, smoking, alcohol consumption, physical activity level, and socioeconomic status. BMI showed the greatest contribution in predicting all-cause mortality, and WWI showed the greatest contribution in predicting CVD mortality. According to the C statistics, the diagnostic model including WWI distinguished the survival times of survivors and decedents by 85% for all-cause mortality and 89% for CVD mortality. The C-statistics showed that all adiposity indices were comparable; however, the order is the same as the goodness of fit statistics.

**Table 4:** Likelihood ratio and C statistics for each adiposity index in diagnostic models* for mortality. (values in parentheses are standard errors).

	Marginal effect				Joint effect with BMI			
	All-cause mortality		CVD mortality		All-cause mortality		CVD mortality	
	LR	C	LR	C	LR	C	LR	C
BMI	960	0.850 (0.011)	38	0.886 (0.03)	—	—	—	—
WC	608	0.848 (0.012)	25^a^	0.886 (0.03)	843.9	0.851 (0.012)	56.8	0.886 (0.03)
WHtR	352	0.848 (0.012)	13^b^	0.886 (0.03)	915.9	0.851 (0.011)	66.8	0.887 (0.03)
ABSI	627	0.850 (0.011)	43	0.886 (0.03)	1162.2	0.852 (0.011)	73.8	0.887 (0.029)
WWI	505	0.849 (0.012)	48	0.887 (0.03)	1176.4	0.852 (0.011)	89.4	0.888 (0.029)

^*^Adjusted by age, sex, systolic blood pressure, fasting glucose, hemoglobin, alanine aminotransferase, gamma-glutamyl transferase, smoking, alcohol, physical activity and socioeconomic status.

LR, likelihood ratio. The p-values of ^a^ and ^b^ for LR are 0.0037 and 0.163, respectively, and all p-values of the other LRs are less than 0.0001.

Because BMI provided the strongest discriminatory power for all-cause mortality, and differs from other measures in that it does not include waist measurements, we tested which measure was the most complementary to BMI in predicting all-cause and CVD mortality. Table 4 shows that WWI is the best complementary indicator of BMI for all-cause and CVD mortality. This implies that WWI possesses the most independent information from BMI to describe the risk of all-cause and CVD mortality.

Figure 1 illustrates the hazard ratios of all-cause and CVD mortality by 10-group stratifications for each adiposity index when each index was included alone in the model (marginal model, Supplementary Tables 1 and 2 for confidence intervals of hazard ratios), and together with BMI (joint model, Supplementary Fig. 1 and Tables 4 and 5). First of all, we observed that BMI was not positively associated with all-cause and cardiovascular mortality, indicating the obesity paradox. Second, WC and WHtR were also not positively associated with all-cause mortality in the marginal model as BMI did, whereas, in contrast, they were positively associated in the joint model. These results originated from the high correlation between BMI and WC or WHtR, as shown in Table 3. Third, WWI and ABSI were positively associated with all-cause and cardiovascular mortality for both marginal and joint models.

**Table 5:** Likelihood ratio for adiposity index, IAUC, and C statistics in prognostics models* for mortality. (values in parentheses are standard errors).

	Joint effect	LR	AUC			IAUC	C
			at year 1	at year 3	at year 5		
All-cause mortality	BMI + WC	264	0.89 (0.015)	0.869 (0.006)	0.865 (0.004)	0.868	0.865 (0.003)
	BMI + WHtR	314	0.891 (0.015)	0.862 (0.006)	0.866 (0.004)	0.869	0.868 (0.003)
	BMI + ABSI	338	0.892 (0.015)	0.862 (0.006)	0.866 (0.004)	0.869	0.867 (0.004)
	BMI + WWI	338	0.891 (0.015)	0.862 (0.006)	0.866 (0.004)	0.869	0.867 (0.004)
CVD mortality	BMI + WC	35	0.938 (0.013)	0.906 (0.011)	0.909 (0.008)	0.912	0.901 (0.007)
	BMI + WHtR	60	0.934 (0.014)	0.909 (0.01)	0.912 (0.007)	0.912	0.904 (0.007)
	BMI_ABSI	46	0.937 (0.013)	0.907 (0.011)	0.911 (0.008)	0.913	0.903 (0.008)
	BMI + WWI	65	0.938 (0.014)	0.909 (0.01)	0.912 (0.008)	0.914	0.905 (0.01)

^*^Adjusted by age and sex and all LRs are significant with p-value < 0.001.

### Prognostic prediction model

Tables 5 and 6 provided LR, time-dependent AUC with its standard error, IAUC that is average of AUCs for entire study time period, and C statistics from prognostic models with different adiposity indices. It appeared that the BMI plus WWI model had the best performance in model fit and predictive accuracy for the risks of all-cause and CVD mortality. This confirmed the result in diagnostic prediction model where WWI was the best complement for BMI. Regardless of models, AUC was the largest at year 1, the smallest at year 3, and between at other years because IAUC was in between. This implies that the predictive accuracy of each model hovered around its respective level of IAUC until the censoring time (i.e., 2013). The models for the risk of all-cause mortality had lower predictive accuracy than those for the risk of CVD mortality. The hazard ratios of all-cause and CVD mortality by 10-group stratifications for each adiposity index in prognostic models were essentially the same patterns as in diagnostic models.

**Table 6:** Likelihood ratio for each adiposity index in diagnostic models* for incident cardiometabolic diseases.

	Marginal effects			Joint effects with BMI		
	Hypertension	Type 2 diabetes	CVD	Hypertension	Type 2 diabetes	CVD
BMI	15972	14070	10444	—	—	—
WC	15439	5744	2309	18532	6184	2567
WHtR	14988	5120	2061	18667	5907	2463
ABSI	4382	1281	691	18578	6343	2580
WWI	6798	12680	10132	18799	16490	11725

^*^Adjusted by age and sex, all LRs are significant with p-values < 0.0001.

### Adiposity index and risk of cardiometabolic diseases

LR test statistics of diagnostic prediction models for marginal and joint effects of adiposity indices on the risks of incident hypertension, type 2 diabetes and CVD are displayed in the Table 6. When each of the indices were included solely in the model, BMI was the best in predicting all the three types of cardiometabolic diseases, while WWI was the second best for incident type 2 diabetes and CVD, and WC was the second best for hypertension. Nevertheless, when each indicator was included together with BMI in the model, WWI as a complementary indicator of BMI was the best in predicting all three types of cardiometabolic diseases.

**Figure 2:**
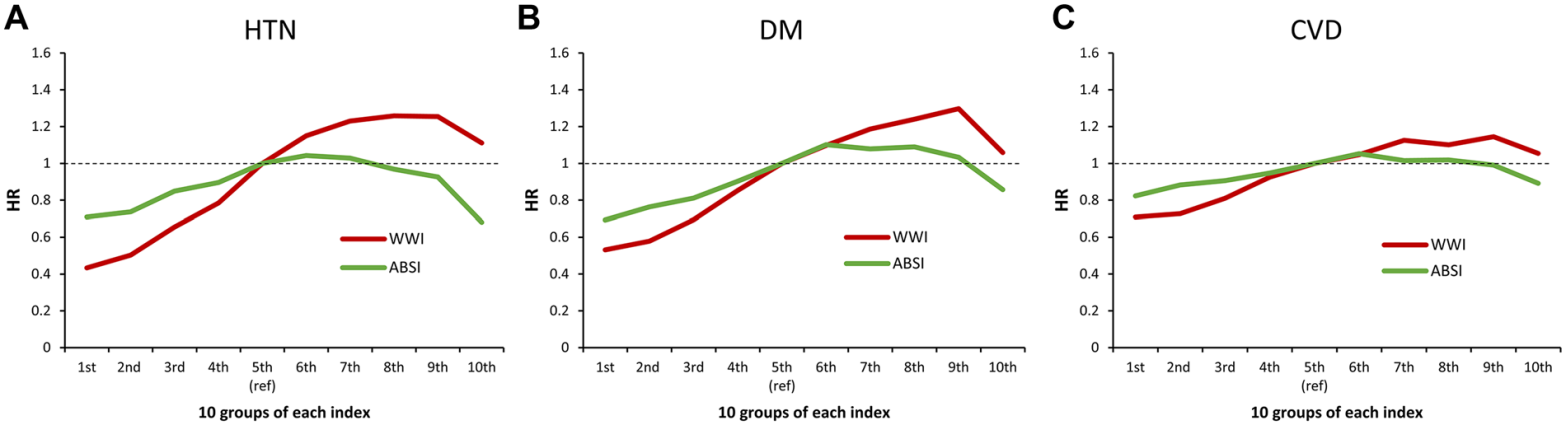
Hazard ratio of 10 groups of each adiposity index on incident hypertension (**A**) type 2 diabetes (**B**) and cardiovascular disease (**C**). (marginal effect).

Figure 2 and Supplementary Fig. 2 depict the hazard ratios of incident cardiometabolic diseases for WWI and ABSI calculated by the marginal and joint models (Supplementary Tables 3 and 6 for confidence intervals of hazard ratios). The trend of hazard ratios of ABSI clearly depends on whether it was adjusted for BMI or not. ABSI was not positively associated with incident hypertension in the marginal model but was positively associated with it in the joint model, implying that ABSI should be adjusted by BMI to evaluate its effect on hypertension. On the other hand, WWI was positively associated with hypertension and hence was independent of BMI in measuring the risk of hypertension. For type 2 diabetes and CVD, a similar pattern was observed (Supplementary Fig. 2).

## Discussion

In this study, we proposed a new adiposity index, WWI, as an integrated predictor of both cardiometabolic disease morbidity and mortality. From the validation process, we proved that WWI has a good predictive ability for both cardiometabolic morbidity and mortality in the Korean population. In addition, WWI has a positive association with all the outcomes, which was not shown in BMI and WC.

Given the increasing prevalence of obesity and obesity-related disorders in the modern society, it is critical to assess obesity and to identify individuals at risk of cardiometabolic diseases in clinical practice. Among all the anthropometric measures of obesity, BMI has been the most widely-used anthropometric indicator due to its simple calculation and good performance in predicting cardiometabolic disease risk. In our analysis, BMI was also proven to have better discriminatory power especially for all-cause mortality and prediction ability for hypertension, type 2 diabetes, and CVD than other indicators. However, along with some reports from other populations, we observed a U-shaped pattern of association between BMI and all-cause, and CVD mortality in our previous study of the Korean population^6^. We also identified that the “obesity paradox” phenomenon was intensified in that the BMI range of the lowest mortality has been shifted from 23–25 kg/m^2^ (overweight) to 25–29.9 kg/m^2^ (moderate obesity) during the last 10 years. These results partly suggested that BMI was limited as a true measure of obesity. In addition, the U-shaped, or J-shaped pattern of the association has frequently been observed in the Asian population whose BMI values were generally lower than those of Caucasians^27–29^. A previous study provided some clues for this difference in that BMI is largely limited to assess for adiposity (fat mass) particularly among individuals with BMI ≤ 30 kg/m^2^ ^30^.

On the other hand, WC was proposed as an alternative measure of obesity, especially for central obesity. With its closer association with visceral adiposity than BMI, it has been suggested as an indicator of metabolic obesity in a lot of studies^31,32^. However, we also found an inverse association between WC and all-cause, CVD mortality in our study population, indicating the limitation of WC as a BMI-dependent index.

These observations strongly required a more accurate, clinically applicable indicator for assessing obesity. Therefore, we produced a novel adiposity index more closely associated with obesity-related disease morbidity and mortality. When the BMI was first proposed, it was calculated by standardizing weight for height^25^. With the same concept, we proposed an index which standardizes waist circumference for body weight. As a result, this new indicator showed expected positive associations with all-cause and CVD mortality unlike BMI, WC, and WHR, expected positive associations with hypertension, type 2 diabetes, and CVD unlike ABSI, and was the best in predicting the risks of all-cause and CVD deaths and of cardiometabolic disease onsets when it was used together with BMI.

There are some limitations to this study. Although our study focused on identifying a new adiposity index as a predictor for the risks of death and diseases, the WWI was developed and validated with the same dataset and, therefore, there is a risk of overfitting and optimism in evaluation of the predictive performance^33^. Hence, we need to include some forms of internal and external validations for the predictive performance of WWI. The variability of WC measurement possibly limits the reliability of the observed association between this WC-based index and outcomes. We did not obtain information about inter-rater or inter-individual variability of WC measurements because we used given data from the established cohort. However, measurement of WC in the Korean national health check-up programs were conducted by trained instructors in each health check-up centers, and the measurement protocol indicates that WC should be measured at the midpoint between the lower rib margin and the iliac crest in the standing position. In addition, we were not able to test the direct relationship between WWI and visceral or subcutaneous adipose tissue area because of lack of data. We also are not sure that this index would be applicable to other population. Further studies using abdominal fat quantification by computed tomography imaging, or for other population would provide the more useful information in this regard.

In conclusion, we propose a novel adiposity index, WWI, as a useful alternative marker of obesity and obesity-related adverse health consequences. In addition, when WWI and BMI are combined, it has the best performance for the prediction of cardiometabolic disease and mortality. It also involves a simple calculation and easy interpretation. So far, the known measures of obesity do not predict both morbidity and mortality with linear trends. Thus, this new index is expected to provide easy information of individuals at risk of cardiometabolic disease and associated mortality with just one measure.

## Electronic supplementary material

Supplementary data

## Data Availability

The data are available for replication through approval and oversight by the Korean National Health Insurance Service (NHIS).
