# Supplementary material for: A novel adiposity index as an integrated predictor of cardiometabolic disease morbidity and mortality
Source: Sci Rep. 2018 Nov 13;8:16753. doi: 10.1038/s41598-018-35073-4 (PMC6233180; doi:10.1038/s41598-018-35073-4)
Supplement: Supplementary file 1 — Supplementary data [file 41598_2018_35073_MOESM1_ESM.pdf]

A novel adiposity index as an integrated predictor of cardiometabolic disease morbidity and mortality

Yousung Park[1*],Nam Hoon Kim[2*], Tae Yeon Kwon[3], Sin Gon Kim[2]

[1]Department of Statistics, Korea University, Seoul, South Korea

[2]Division of Endocrinology and Metabolism, Department of Internal Medicine, Korea University College of Medicine, Seoul, South Korea

[3]Department of International Finance, Hankuk University of Foreign Studies, Yongin-si, Gyeonggi-do, South Korea

Supplementary Figure 1. Hazard ratio of 10 groups of each adiposity index on all-cause and on all-cause and cardiovascular mortality when each index was included with BMI in the model (joint effect)

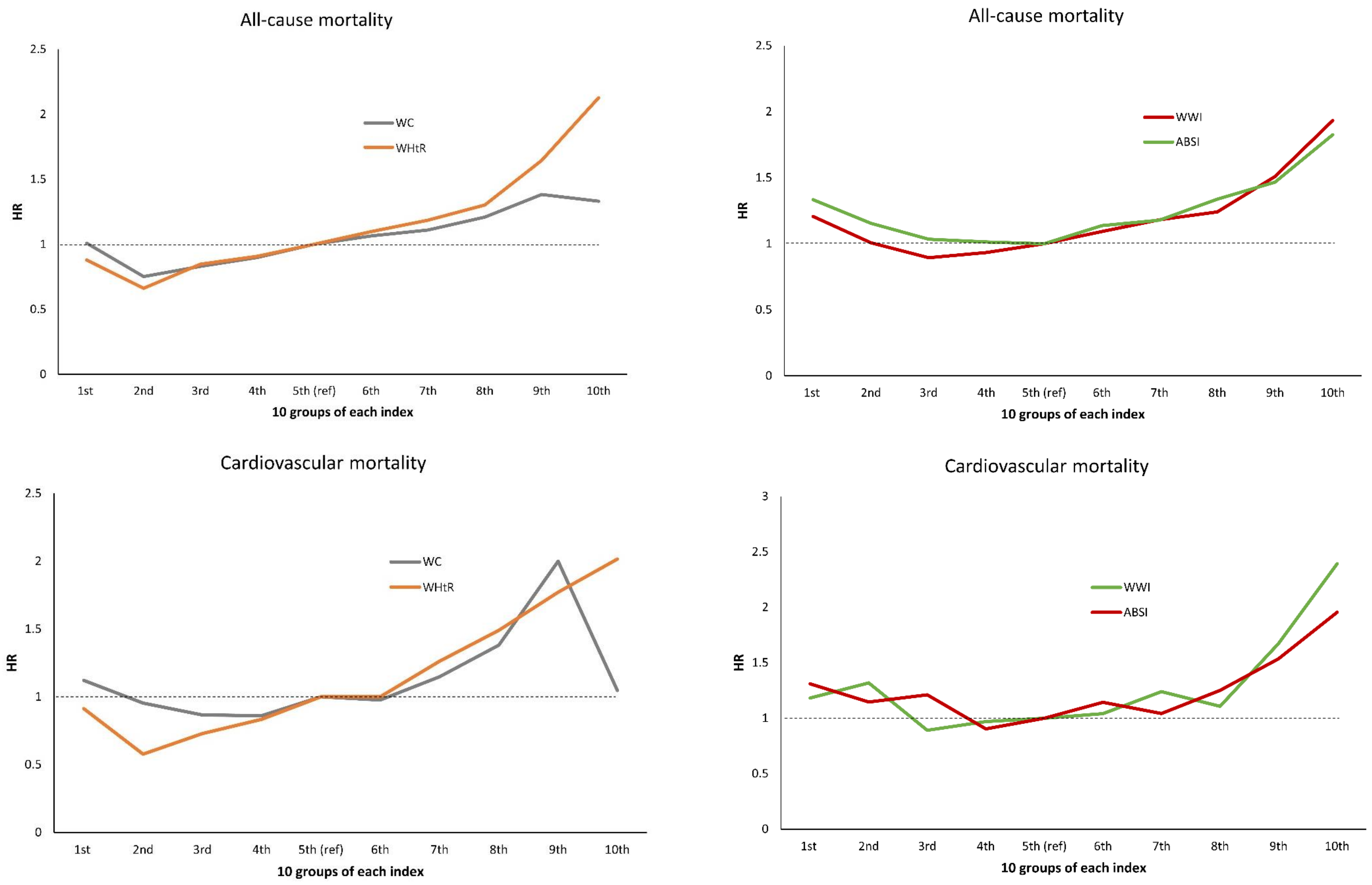

Supplementary Figure 2. Hazard ratio of 10 groups of each adiposity index on incident hypertension, type 2 diabetes and cardiovascular disease when each index was included with BMI in the model (joint effect).

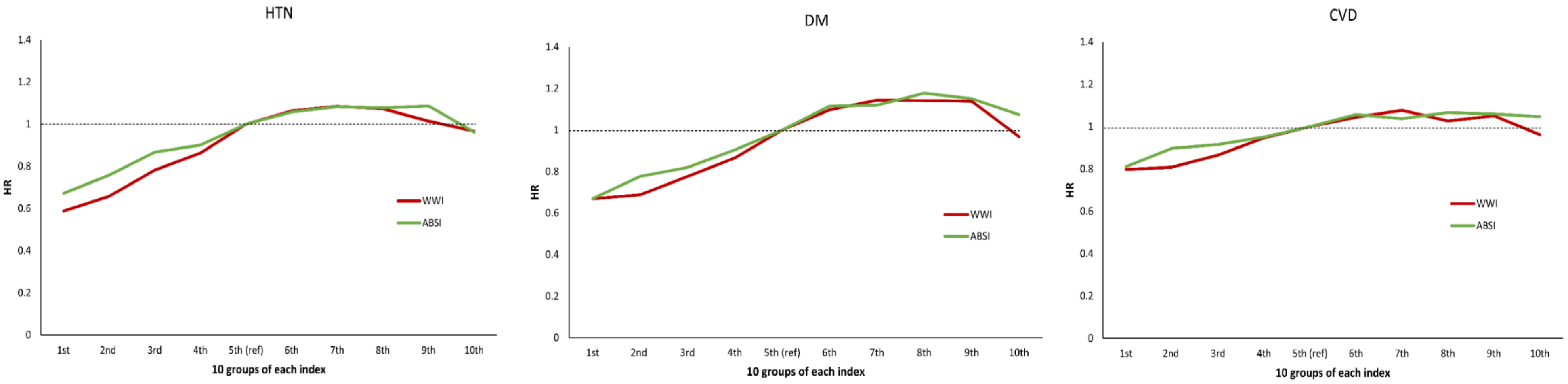

Supplementary Table 1. Hazard ratios and 95% confidence intervals of adiposity indices for all-cause mortality (marginal effect)

Index		1st	2nd	3rd	4th	5th	6th	7th	8th	9th	10th
BMI	HR	2.568	1.887	1.577	1.175	1	0.935	0.843	0.856	0.813	1.423
	95% CI lower limit	2.328	1.72	1.45	1.081	1	0.851	0.753	0.746	0.664	1.092
	95% CI upper limit	2.832	2.071	1.715	1.277	1	1.027	0.944	0.981	0.995	1.855
WC	HR	2.244	1.459	1.332	1.156	1	0.891	0.826	0.815	0.868	0.865
	95% CI lower limit	1.897	1.291	1.217	1.069	1	0.822	0.753	0.732	0.745	0.693
	95% CI upper limit	2.654	1.649	1.458	1.249	1	0.967	0.905	0.909	1.01	1.08
WHtR	HR	1.994	1.267	1.355	1.166	1	0.89	0.837	0.81	0.885	1.028
	95% CI lower limit	1.662	1.115	1.215	1.073	1	0.822	0.759	0.738	0.776	0.888
	95% CI upper limit	2.392	1.441	1.512	1.268	1	0.963	0.923	0.889	1.011	1.189
WWI	HR	1.532	1.261	1.049	1.017	1	1.023	1.063	1.089	1.309	1.793
	95% CI lower limit	1.16	1.041	0.911	0.911	1	0.941	0.972	0.993	1.18	1.593
	95% CI upper limit	2.025	1.526	1.208	1.134	1	1.113	1.164	1.195	1.452	2.019
ABSI	HR	1.372	1.205	1.063	1.024	1	1.128	1.187	1.372	1.562	2.257
	95% CI lower limit	1.073	1.005	0.931	0.917	1	1.032	1.082	1.255	1.411	2.023
	95% CI upper limit	1.755	1.444	1.213	1.142	1	1.233	1.303	1.5	1.729	2.517

Supplementary Table 2. Hazard ratios and 95% confidence intervals of adiposity indices for cardiovascular mortality (marginal effect)

Index		1st	2nd	3rd	4th	5th	6th	7th	8th	9th	10th
BMI	HR	2.129	1.273	1.287	1.045	1	0.976	1.125	1.075	0.74	0.958
	95% CI lower limit	1.629	0.966	1.018	0.831	1	0.76	0.853	0.763	0.411	0.392
	95% CI upper limit	2.782	1.677	1.628	1.314	1	1.254	1.485	1.514	1.331	2.339
WC	HR	1.992	1.502	1.169	1.003	1	0.884	0.967	1.089	1.437	0.673
	95% CI lower limit	1.258	1.085	0.903	0.802	1	0.703	0.757	0.829	1.016	0.331
	95% CI upper limit	3.154	2.08	1.512	1.255	1	1.111	1.235	1.431	2.032	1.369
WHtR	HR	1.867	1.004	1.068	1.012	1	0.857	0.99	1.068	1.125	1.094
	95% CI lower limit	1.109	0.674	0.762	0.791	1	0.684	0.765	0.842	0.811	0.75
	95% CI upper limit	3.143	1.494	1.498	1.296	1	1.074	1.28	1.353	1.56	1.596
WWI	HR	1.328	1.489	0.975	1.017	1	1.004	1.173	1.031	1.542	2.328
	95% CI lower limit	0.54	0.867	0.628	0.733	1	0.785	0.911	0.791	1.17	1.724
	95% CI upper limit	3.265	2.558	1.514	1.411	1	1.285	1.51	1.344	2.033	3.145
ABSI	HR	1.306	1.163	1.226	0.908	1	1.137	1.041	1.259	1.573	2.191
	95% CI lower limit	0.634	0.678	0.853	0.656	1	0.888	0.799	0.983	1.2	1.647
	95% CI upper limit	2.69	1.996	1.76	1.258	1	1.457	1.358	1.611	2.061	2.916

Supplementary Table 3. Hazard ratios and 95% confidence intervals of adiposity indices for diseases of HTN, DM, and CVD (marginal effect)

Disease	Index		1st	2nd	3rd	4th	5th	6th	7th	8th	9th	10th
HTN	WWI	HR	0.434	0.503	0.655	0.785	1	1.15	1.23	1.258	1.255	1.111
		95% CI lower limit	0.399	0.479	0.634	0.765	1	1.125	1.2	1.225	1.213	1.06
		95% CI upper limit	0.472	0.528	0.676	0.806	1	1.175	1.26	1.292	1.298	1.164
	ABSI	HR	0.709	0.737	0.849	0.896	1	1.042	1.029	0.968	0.926	0.681
		95% CI lower limit	0.668	0.708	0.825	0.875	1	1.019	1.003	0.943	0.893	0.648
		95% CI upper limit	0.752	0.766	0.873	0.918	1	1.065	1.055	0.994	0.959	0.716
DM	WWI	HR	0.532	0.579	0.695	0.853	1	1.097	1.186	1.24	1.297	1.059
		95% CI lower limit	0.486	0.553	0.672	0.828	1	1.067	1.152	1.203	1.246	1.006
		95% CI upper limit	0.581	0.606	0.719	0.879	1	1.128	1.222	1.279	1.352	1.114
	ABSI	HR	0.693	0.764	0.811	0.903	1	1.101	1.079	1.09	1.033	0.857
		95% CI lower limit	0.648	0.731	0.785	0.878	1	1.073	1.047	1.057	0.991	0.809
		95% CI upper limit	0.742	0.8	0.838	0.929	1	1.13	1.111	1.125	1.076	0.909
CVD	WWI	HR	0.709	0.727	0.81	0.925	1	1.047	1.125	1.101	1.145	1.054
		95% CI lower limit	0.642	0.69	0.78	0.894	1	1.015	1.089	1.066	1.097	1.001
		95% CI upper limit	0.785	0.766	0.841	0.956	1	1.08	1.162	1.138	1.196	1.11
	ABSI	HR	0.823	0.883	0.906	0.947	1	1.052	1.015	1.018	0.991	0.892
		95% CI lower limit	0.762	0.84	0.873	0.917	1	1.022	0.982	0.985	0.949	0.842
		95% CI upper limit	0.888	0.929	0.941	0.978	1	1.083	1.048	1.053	1.035	0.944

Supplementary Table 4. Hazard ratios and 95% confidence intervals of adiposity indices for all-cause mortality when each index was included with BMI in the model (joint effect).

Index		1st	2nd	3rd	4th	5th	6th	7th	8th	9th	10th
WC	HR	1.009	0.752	0.831	0.899	1	1.064	1.11	1.209	1.383	1.331
	95% CI lower limit	0.837	0.655	0.752	0.829	1	0.978	1.004	1.069	1.16	1.023
	95% CI upper limit	1.215	0.863	0.919	0.975	1	1.157	1.227	1.368	1.648	1.732
WHtR	HR	0.879	0.662	0.847	0.908	1	1.098	1.186	1.303	1.645	2.127
	95% CI lower limit	0.72	0.575	0.754	0.833	1	1.012	1.068	1.173	1.415	1.779
	95% CI upper limit	1.072	0.762	0.952	0.99	1	1.192	1.316	1.449	1.912	2.544
WWI	HR	1.207	1.008	0.894	0.934	1	1.093	1.182	1.241	1.509	1.933
	95% CI lower limit	0.912	0.832	0.776	0.837	1	1.004	1.08	1.131	1.36	1.717
	95% CI upper limit	1.596	1.221	1.03	1.042	1	1.189	1.294	1.362	1.675	2.177
ABSI	HR	1.334	1.155	1.035	1.014	1	1.137	1.179	1.338	1.466	1.825
	95% CI lower limit	1.043	0.963	0.907	0.908	1	1.04	1.075	1.224	1.324	1.634
	95% CI upper limit	1.707	1.385	1.182	1.131	1	1.243	1.294	1.463	1.623	2.04

Supplementary Table 5. Hazard ratios and 95% confidence intervals of adiposity indices for cardiovascular mortality when each index was included with BMI in the model (joint effect).

Index		1st	2nd	3rd	4th	5th	6th	7th	8th	9th	10th
WC	HR	1.121	0.954	0.866	0.861	1	0.977	1.148	1.381	2	1.048
	95% CI lower limit	0.673	0.658	0.651	0.681	1	0.772	0.88	1.01	1.33	0.48
	95% CI upper limit	1.866	1.381	1.153	1.087	1	1.237	1.497	1.887	3.008	2.288
WHtR	HR	0.913	0.577	0.729	0.834	1	1	1.263	1.491	1.771	2.016
	95% CI lower limit	0.52	0.376	0.511	0.647	1	0.793	0.96	1.137	1.219	1.282
	95% CI upper limit	1.6	0.887	1.042	1.075	1	1.261	1.661	1.957	2.574	3.169
WWI	HR	1.182	1.319	0.891	0.97	1	1.041	1.24	1.107	1.673	2.393
	95% CI lower limit	0.48	0.766	0.573	0.699	1	0.813	0.961	0.848	1.266	1.77
	95% CI upper limit	2.911	2.27	1.386	1.347	1	1.333	1.598	1.446	2.21	3.234
ABSI	HR	1.31	1.146	1.211	0.904	1	1.144	1.043	1.251	1.536	1.955
	95% CI lower limit	0.636	0.667	0.843	0.653	1	0.893	0.8	0.977	1.171	1.462
	95% CI upper limit	2.7	1.967	1.74	1.251	1	1.466	1.359	1.603	2.014	2.615

Supplementary Table 6. Hazard ratios and 95% confidence intervals of adiposity indices of HTN, DM, and CVD when each index was included with BMI in the model (joint effect).

Disease	Index		1st	2nd	3rd	4th	5th	6th	7th	8th	9th	10th
HTN	WWI	HR	0.588	0.658	0.782	0.863	1	1.063	1.085	1.073	1.015	0.967
		95% CI lower limit	0.54	0.627	0.757	0.841	1	1.04	1.058	1.045	0.981	0.922
		95% CI upper limit	0.639	0.692	0.808	0.886	1	1.086	1.112	1.102	1.05	1.013
	ABSI	HR	0.672	0.758	0.867	0.901	1	1.058	1.083	1.077	1.087	0.963
		95% CI lower limit	0.634	0.729	0.843	0.879	1	1.035	1.056	1.049	1.048	0.916
		95% CI upper limit	0.713	0.788	0.892	0.923	1	1.082	1.11	1.106	1.126	1.013
DM	WWI	HR	0.669	0.688	0.776	0.866	1	1.097	1.144	1.142	1.139	0.968
		95% CI lower limit	0.614	0.652	0.747	0.84	1	1.069	1.111	1.107	1.095	0.915
		95% CI upper limit	0.728	0.725	0.805	0.892	1	1.125	1.178	1.179	1.185	1.025
	ABSI	HR	0.67	0.777	0.82	0.905	1	1.115	1.12	1.177	1.151	1.074
		95% CI lower limit	0.626	0.743	0.793	0.88	1	1.087	1.088	1.141	1.104	1.012
		95% CI upper limit	0.717	0.813	0.847	0.931	1	1.145	1.154	1.214	1.2	1.139
CVD	WWI	HR	0.797	0.809	0.866	0.947	1	1.044	1.077	1.027	1.051	0.962
		95% CI lower limit	0.722	0.761	0.83	0.915	1	1.015	1.043	0.993	1.008	0.909
		95% CI upper limit	0.879	0.859	0.905	0.98	1	1.074	1.112	1.063	1.096	1.018
	ABSI	HR	0.811	0.898	0.916	0.951	1	1.057	1.038	1.067	1.06	1.047
		95% CI lower limit	0.751	0.853	0.883	0.921	1	1.027	1.005	1.033	1.015	0.988
		95% CI upper limit	0.875	0.944	0.951	0.982	1	1.088	1.072	1.103	1.107	1.11
